# Identification of Human Serum Peptides in Fourier Transform Ion Cyclotron Resonance Precision Profiles

**DOI:** 10.1155/2012/804036

**Published:** 2012-05-22

**Authors:** Simone Nicolardi, Hans Dalebout, Marco R. Bladergroen, Wilma E. Mesker, Rob A. E. M. Tollenaar, André M. Deelder, Yuri E. M. van der Burgt

**Affiliations:** ^1^Department of Parasitology, Biomolecular Mass Spectrometry Unit, Leiden University Medical Center (LUMC), Albinusdreef 2, 2300 RC Leiden, The Netherlands; ^2^Department of Surgery, Leiden University Medical Center (LUMC), Albinusdreef 2, 2300 RC Leiden, The Netherlands

## Abstract

The continuous efforts to find new prognostic or diagnostic biomarkers have stimulated the use of mass spectrometry (MS) profiles in a clinical setting. In the early days (about one decade ago), a single low-resolution mass spectrum derived from an individual's body fluid was used for comparative studies. However, a peptide profile of a complex mixture is most informative when recorded on an ultrahigh resolution instrument such as a Fourier transform ion cyclotron resonance (FTICR) mass spectrometer. In this study we show the benefits of the ultrahigh resolving power and the high mass accuracy and precision provided by an FTICR mass spectrometer equipped with a 15-tesla magnet. The ultrahigh-resolution data not only allow assignment of fragment ions with high charge states (4+, 5+) but also enhance confidence of human serum peptide identifications from tandem MS experiments. This is exemplified with collision-induced dissociation (CID) and electron transfer dissociation (ETD) data of middle-down-sized endogenous or protein-breakdown peptides that are of interest in biomarker discovery studies.

## 1. Introduction

Mass spectrometry- (MS-) based proteomics is a principal platform in systems biology, that is, the integrated approach of different technical disciplines to study the physiological processes in a cell or tissue [1–3]. The diagnosis of a disease or monitoring new medicines in a (pre)clinical setting can be performed either through (theoretically) *full* proteome analysis or by molecular profiling of large cohorts of biological samples. Nowadays, the first approach is technically feasible, however at low throughput [4]. In the latter high-throughput approach the relevant differences between numerous peptides and proteins are mapped in affected tissues or body fluids of healthy and diseased individuals [5, 6]. For this purpose differential analyses of protein expression patterns in cells, tissue, or body fluids such as plasma or cerebrospinal fluid (CSF) are performed. These patterns can change as a result of disease and are thus helpful in both early detection and monitoring the development of the disease. Additionally, detection of biomarkers can also play a significant role in prevention. Proteomic approaches have been successfully used to obtain information on the state of protein circuits inside tumor cells and at the tumor-host interface [7]. Moreover, the analysis of peripheral blood (serum samples) for biomarkers has emerged as a promising tool for cancer diagnostics, disease monitoring, and prognostication of the patient. Unfortunately, many biomarker discovery profiling studies have not provided sufficient information on the identity of the peptides or proteins of interest. One reason for this incompleteness is the technical limitation of the used MS-platform with respect to resolving power and MS/MS capabilities. In this work we show the benefits of using high-end Fourier transform ion cyclotron resonance (FTICR) MS for identification of human serum peptides. These benefits are even more clear taking into account that many of these peptides are endogenous or result from protein degradation.

### 1.1. Ultrahigh Resolution and- Precision Provided by FTICR

It is well established that the sub-ppm mass accuracy of fourier transform ion cyclotron resonance mass spectrometry (FTICR-MS) improves the results and increases the confidence of identifications [8, 9]. Previously, we have shown that a combination of highly standardized sample workup protocols with FTICR precision profiles results in very low mass measurement errors (MMEs) [9]. Moreover, FTICR-MS is a powerful tool in tackling sample complexity due to its ultrahigh mass resolution characteristics [10]. Several recent instrumental and software developments (improved electronics and computer technology) have made FTICR-MS accessible to a broader research community, including the life science area and the clinic. With suitable control and proper calibration, high field FTICR-MS routinely achieves an MME lower than 1 ppm [11, 12]. At very high fields, such as in recently available 15-tesla instruments, MMEs at a ppb level can be reached. The confidence in peptide and protein identifications improves significantly with increasing mass accuracy, especially if mass accuracies below 1 ppm are reached. Moreover, an increased mass accuracy assists in the identifications of peptides that are not in a database (e.g., unsequenced species, posttranslational modifications). The potential of sub-ppm mass accuracy allows for in-depth comparisons of complex peptide mixtures obtained from patient and control body fluids. In order to properly use accurate mass values obtained from modern high-end mass analyzers guidelines have been reported with respect to statistical mass accuracy [13].

### 1.2. Tandem MS Using Electron Transfer Dissociation

Peptide sequencing with *electron transfer dissociation* (ETD) MS/MS has proven to be an extremely powerful and complementary tool next to the widely used *collision-induced dissociation *(CID) [14, 15]. In a bottom-up proteomics workflow the peptide sequence coverage is often improved with combined CID and ETD fragmentation, either performed on-line in an alternating way or off-line in a sequential experiment [16, 17]. Initially, ETD was implemented on ion trap mass spectrometers and this is still the most commonly applied platform to perform peptide sequencing experiments. In the field of MS-based proteomics it is now well known that ETD is especially favorable for sequencing larger peptides with higher charge states (i.e., 3+, 4+), for middle-down and top-down proteomics, and for determining the location and identity of posttranslational modifications (PTMs) on peptide backbones [15]. Recent developments have enabled ETD experiments on ultrahigh-resolution mass analyzers such as a time of flight (TOF), Orbitrap, and FTICR [15, 18, 19]. In this work we will present both CID and ETD data obtained from an FTICR system from middle-down-sized peptides that are of interest in biomarker discovery studies.

## 2. Materials and Methods

### 2.1. Sample Collection and Serum Peptide Isolation

The protocols for both the collection of human blood samples and for serum peptide isolation have been described previously [9]. Informed consent was obtained from all patients and the Leiden University Medical Center (LUMC) Medical Ethical Committee approved the studies. Serum peptides were isolated in a fully automated and standardized fashion using reversed-phase (RP) C_18_-functionalized magnetic beads. In this study, 10 *μ*L of commercially available RPC_18_ Dynabeads (Invitrogen, Carlsbad, CA, USA) was used for the analysis of 5 *μ*L serum. The manufacturer's protocol was followed for the activation, wash, and desorption steps of the RPC_18_ beads, with adjustments and optimizations to allow implementation on a 96-channel liquid handling Hamilton STAR plus pipetting robot [9].

### 2.2. ESI-FTICR-MS/MS

The eluates obtained from the RPC_18_-magnetic bead workup were used for MS/MS experiments (see sample collection). To this end, eluates from 96 different samples were pooled and ultrafiltrated using a 30 kDa filter (Amicon Ultra 0.5 mL; Millipore). The filtrate was concentrated by lyophilization before RPC_18_-LC-MS analysis on a splitless nanoLC-Ultra 2D plus system (Eksigent, Dublin, CA USA) equipped with a PepMap C_18_ trap column (300 micrometer internal diameter, 5 millimeter length; Dionex, Sunnyvale, CA USA) and a ChromXP C_18_-analytical column (300 micrometer internal diameter, 15 centimeter length; Eksigent). Here, one minute manual fraction collection was performed at a flow rate of 4 microliter per minute, with a gradient of 4% to 44% acetonitril in 0.05% formic acid buffer. Then, the 35 fractions were analysed using a Bruker 15 tesla solariX FTICR mass spectrometer equipped with a CombiSource, a quadrupole for precursor ion selection, a hexapole collision cell for CID, and an ETD source on a splitted octopole after mixing them with 100 microliter of spray solution (50/50 MeOH/H_2_O 0.1% formic acid) [9]. Direct infusion electrospray ionisation (ESI) experiments were carried out at an infusion rate of 2 microliter per minute. The ion funnels were operated at 100 V and 6.0 V, respectively, with the skimmers at 15 V and 5 V. The trapping potentials were set at 0.60 V and 0.55 V, the analyzer entrance was maintained at –7 V, and side kick technology was used to further optimize peak shape and signal intensity. The required excitation power was 28% with a pulse time of 20.0 *μ*s. MS/MS experiments were performed with the Q at an isolation window of 10 mass units, followed by either CID or ETD and fragment ion mass analysis in the ICR cell. For CID experiments, both the collision energy and the accumulation time in the hexapole collision cell were optimized for each precursor ion. Collision energies varied from 5 to 33 V while the accumulation times varied from 1 to 5 seconds. For ETD experiments the ETD reagent accumulation time was fixed at 400 milliseconds (fluoranthene from negative chemical ionization (NCI) source). The ETD spectrum was optimized by varying the reaction time (from 10 to 100 milliseconds, with an optimum at 50 milliseconds), varying the peptide (analyte) accumulation time (depending on the precursor ion intensity), and modifying the mirror RF amplitude (280−300 Vpp).

### 2.3. Data Analysis

It should be noted that the peptides that were sequenced in this work (obtained from human serum samples) were either endogenous peptides or breakdown products from serum proteins. This implies that any amino acid can be present at the C- and the N-terminus. As a consequence, the interpretation of MS/MS spectra was partly performed manually (de novo) and partly using software from Bruker Daltonics (Data Analysis, BioTools). With a manually determined part of the sequence (sequence tag) a BLAST search was performed. These results were further used to match other parts of the MS/MS data.

## 3. Results and Discussion

Matrix-assisted laser desorption ionization (MALDI) precision profiles of serum eluates were obtained using a state-of-the-art solariX-FTICR system equipped with a 15-tesla magnet [9]. Since the purpose of this study is to report peptide identifications, the discussion of such precision profiles is outside the scope of this work. Nevertheless, it should be emphasized that the accurate mass list of peptides obtained from precision profiles allowed a window of overlap between species observed in MALDI profiles and those determined in ESI spectra lower than 1 ppm. For a complex mixture such as serum peptides this can only be achieved in a single-step analysis (i.e., without the need for LC-separation) using the ultrahigh resolving power of FTICR.

**Figure 1:**
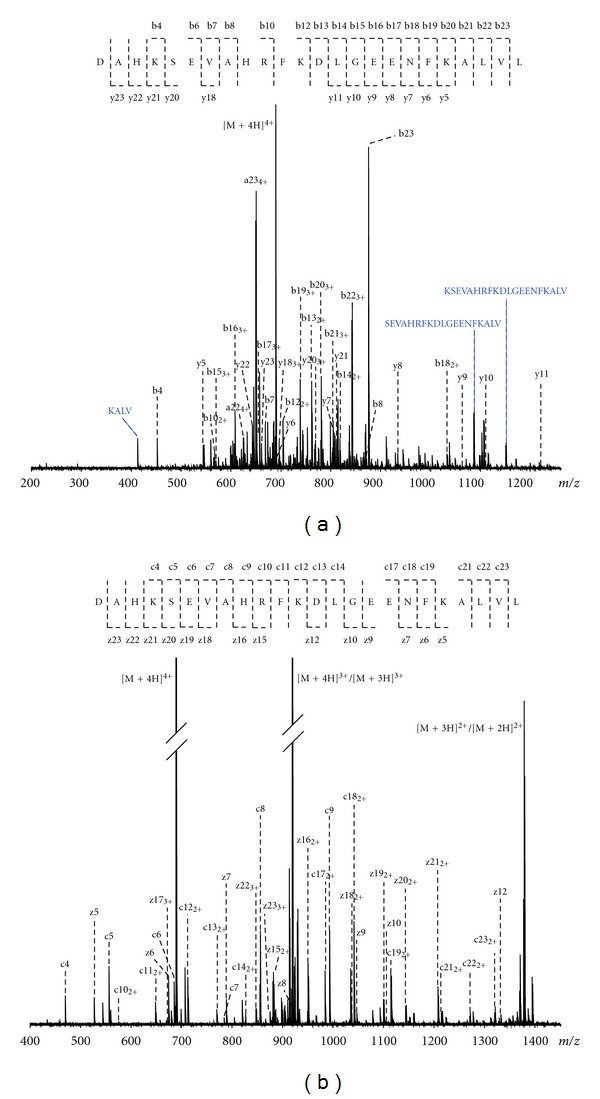
ESI-FTICR-MS/MS spectra of an albumin peptide isolated from a human serum sample, with sequence coverage in CID (a) and ETD (b).

The solariX-FTICR system was further used to perform MS/MS experiments aiming for peptide sequencing. To this end, peptide mixtures were directly infused in ESI mode, isolated in the Q, and fragmented through either CID or ETD. These peptide mixtures were obtained after suitable sample clean-up from the magnetic bead eluates and fractionation (as described in Section 2). As an example, the CID and ETD spectra of an albumin fragment (UniProt P02768, number 25–48) are shown in Figure 1. The ultrahigh resolving power of the FTICR allowed confident assignment of many fragment ions, resulting in a peptide sequence coverage of 87% (20 out of 23 peptide bonds) and 91% from CID and ETD, respectively. From Figure 1 is follows that in the albumin peptide three bond cleavages were not observed upon CID, namely, between the amino acids S-E, H-R, and F-K. In the ETD spectrum these bond cleavages, among others, were observed, stressing the complementarity of these two fragmentation methods. The complementarity is also valid vice versa; namely, bond cleavages between amino acids E-E and K-A were observed in CID and not in ETD. Furthermore, it is interesting to report on the signal intensities of the CID and ETD fragments of the peptide with MW 2754 Da: these varied from 4 × 10*E*6 to 4 × 10*E*8, with a precursor ion intensity of 5 × 10*E*8, and from 1 × 10*E*7 to 3 × 10*E*8, with a precursor ion intensity of 3 × 10*E*9, respectively. This exemplifies the large dynamic range of the FTICR; that is, also the low abundant fragment ions are clearly detectable in an MS/MS spectrum.

**Figure 2:**
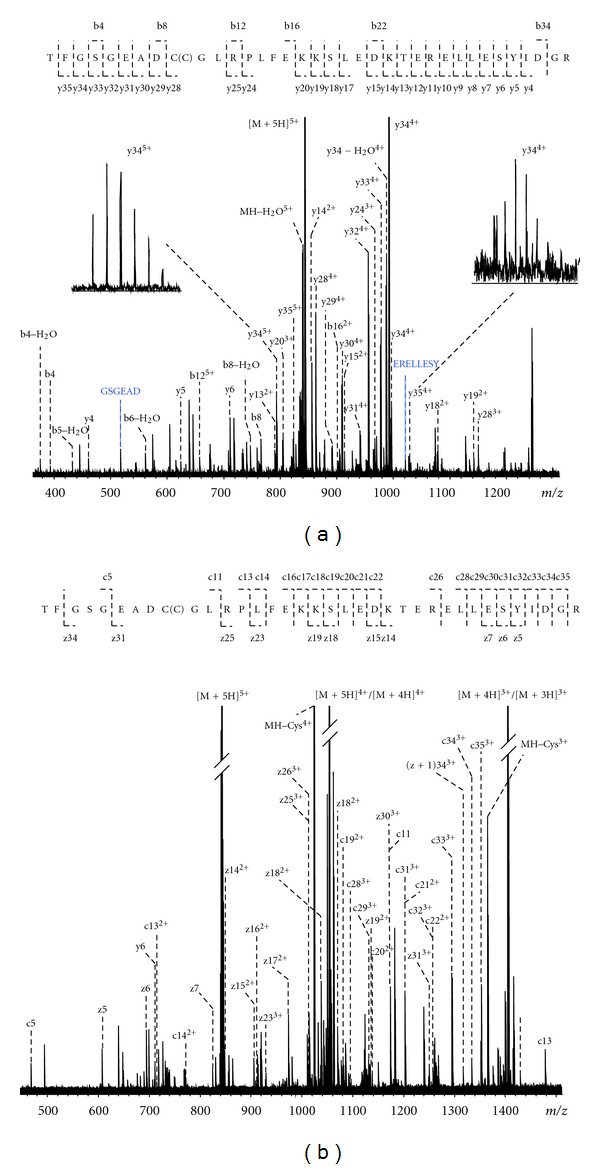
ESI-FTICR-MS/MS spectra of a prothrombin peptide isolated from a human serum sample, with sequence coverage in CID (a) and ETD (b). In order to exemplify the ultrahigh resolving power of the system the insets show the isotopic patterns of fragment ions at *m/z* 793 [y34(5+)] and at *m/z* 1028 [y35(4+)].

**Figure 3:**
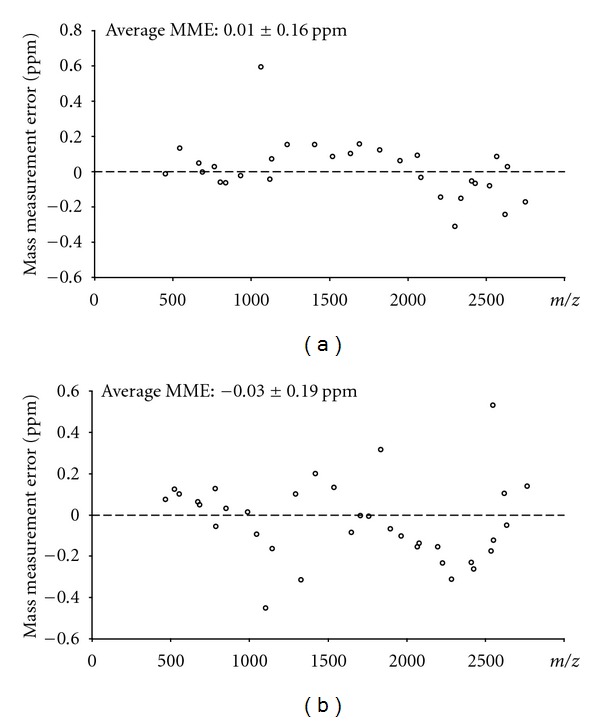
Summary of mass measurement errors of fragment ions in ESI-FTICR-MS/MS CID (a) and ETD (b) spectra of a prothrombin peptide isolated from a human serum sample.

The second example of complementarity of CID and ETD fragmentation is presented in Figure 2, although not up to a total sequence coverage of 100%. The sequence coverage of the prothrombin peptide was 77% from the CID spectrum and 60% from the ETD-spectrum, whereas the combination of data resulted in 89%. This peptide was identified as a fragment from prothrombin (UniProt P00734, number 328–363), namely, TFGSGEADC(C)GLRPLFEKKSLEDKTERELLESYIDGR. Note that this peptide contains a cysteinylation at cysteine 336 (indicated between parentheses) that originates from the disulfide bridge to cysteine 482. Both the ultrahigh resolving power and the sub-ppm MMEs of *all *fragment ions provided by the FTICR were essential for confident assignments. In the case of the CID spectrum especially, the charge states of most fragment ions obtained from the large precursor peptide with five protons could not have been resolved with relatively low-resolution instrumentation such as an ion trap mass spectrometer. This is exemplified by the insets in Figure 2, showing the isotopic patterns of fragment ions at *m/z* 793 [y34(5+)] and at *m/z* 1028 [y35(4+)]. The MMEs are overviewed in Figure 3. The average MMEs for the CID and ETD experiments of the peptide with MW 4210 Da were 0.01 ± 0.16 ppm and −0.03 ± 0.19 ppm, respectively. Again, the benefits of the large dynamic range (more than two orders of magnitude) of detecting fragment ions become clear from the following number: the signal intensities of the CID fragments of the peptide with MW 4210 Da varied from 2.5 × 10*E*6 to 5 × 10*E*8, with a precursor ion intensity of 2 × 10*E*8, and the signal intensities of the ETD fragments of this peptide varied from 1 × 10*E*7 to 6 × 10*E*8, with a precursor ion intensity of 8 × 10*E*9.
